# Angular and linear measurements of adult flexible flatfoot via weight-bearing CT scans and 3D bone reconstruction tools

**DOI:** 10.1038/s41598-021-95708-x

**Published:** 2021-08-09

**Authors:** Maurizio Ortolani, Alberto Leardini, Chiara Pavani, Silvia Scicolone, Mauro Girolami, Roberto Bevoni, Giada Lullini, Stefano Durante, Lisa Berti, Claudio Belvedere

**Affiliations:** 1 Movement Analysis Laboratory, IRCCS Istituto Ortopedico Rizzoli, Via di Barbiano 1/10, Bologna, Italy; 2 Bentivoglio Orthopaedic Ward, IRCCS Istituto Ortopedico Rizzoli, Bologna, Italy; 3 Nursing, Technical and Rehabilitation Assistance Service, IRCCS Istituto Ortopedico Rizzoli, Bologna, Italy

**Keywords:** Musculoskeletal system, Bone imaging, Three-dimensional imaging

## Abstract

Acquired adult flatfoot is a frequent deformity which implies multiple, complex and combined 3D modifications of the foot skeletal structure. The difficult thorough evaluation of the degree of severity pre-op and the corresponding assessment post-op can now be overcome by cone-beam (CBCT) technology, which can provide access to the 3D skeletal structure in weight-bearing. This study aims to report flatfoot deformities originally in 3D and in weight-bearing, with measurements taken using two different bone segmentation techniques. 21 such patients, with indication for surgical corrections, underwent CBCT (Carestream, US) while standing on one leg. From these scans, 3D models of each bone of the foot were reconstructed by using two different state-of-the-art segmentation tools: a semi-automatic (Mimics Innovation Suite, Materialise, Belgium), and an automatic (Bonelogic Ortho Foot and Ankle, Disior, Finland). From both reconstructed models, Principal Component Analysis was used to define anatomical reference frames, and original foot and ankle angles and other parameters were calculated mostly based on the longitudinal axis of the bones, in anatomical plane projections and in 3D. Both bone model reconstructions revealed a considerable valgus of the calcareous, plantarflexion and internal rotation of the talus, and typical Meary’s angles in the lateral and transverse plane projections. The mean difference from these angles between semi-automatic and automatic segmentations was larger than 3.5 degrees for only 3 of the 32 measurements, and a large number of these differences were not statistically significant. CBCT and the present techniques for bone shape reconstruction finally provide a novel and valuable 3D assessment of complex foot deformities in weight-bearing, eliminating previous limitations associated to unloaded feet and bidimensional measures. Corresponding measurements on the bone models from the two segmentation tools compared well. Other more representative measurements can be defined in the future using CBCT and these techniques.

## Introduction

Acquired adult flatfoot, or pes planovalgus, is known to be a frequent deformity including valgus of the hindfoot, flattening of the medial longitudinal arch and abduction of the forefoot^1–3^. It implies multiple, complex and combined three-dimensional (3D) modifications of the foot bone geometry and alignments, both in static and dynamic^4–6^ conditions. To evaluate the degree of severity, to make clinical indication and to assess quantitatively the outcomes of treatments, both conservative or surgical, thorough measurements of all these deformities are necessary in support to the standard clinical assessment^6,7^. In particular, it has been recently recommended^6^ for treatment decisions to be more individualized to each patient’s deformity, this being complex, 3D, and involving hindfoot, midfoot and forefoot. Because these quantitative assessments of the foot have a value only in weight-bearing, these measurements have been performed for decades in lateral or dorsal x-ray pictures in up-right postures^7^, resulting also in an overabundance of similar angles^8,9^. These are two-dimensional by their nature, thus missing the important 3D character of most of the flatfoot deformities, in addition to the limits of bones superimposition and lack of reproducibility^10^. This is particularly true in the flatfoot for the subtalar joint^11^. On the other hand, the alternative 3D reconstructions of foot bones in supine from standard computed tomography (CT) is limited by the unloading conditions of the scans^12,13^. Specialized devices were constructed to apply vertical load to the feet during CT examination in supine^14–16^, but the complex physiological weight-bearing condition of the legs is not reproduced. Also, multiplanar imaging was proposed^11,17–19^; though in weight-bearing, this still implied planar and not fully 3D measures.

Recently, cone-beam technology for CT (CBCT) scans has overcome the critical traditional option between two-dimensional medical imaging under load from simple radiographs, and 3D scans in unloaded conditions of the traditional CT, i.e. where scans are collected with patients lying in supine position. These modern CBCT devices can provide 3D scans in weight-bearing, which is particularly valuable for foot and ankle studies^20–24^. This new feature adds to the demonstrated relatively low radiation, high spatial resolution, and convenient ergonomy and post-processing^13,25–29^, making this technology fundamental nowadays in many clinical studies^6,7,10,12–16,20–23,26–28,30^. The load can be modulated from case to case, ranging from standard single- or double-leg up-right postures to other postural conditions with the leg under determined and controlled positions^13^. The direct effect on skeletal structures of shoes and orthotics can also be assessed straightaway. Even the overall acquisition time and costs of the radiological assessments are claimed to be smaller^31^.

However, any thorough quantitative analysis of the foot’s overall skeletal geometry from CT requires careful reconstruction of 3D bone models starting from the series of image slices. Typically, in each image the contour of each relevant bone has to be identified and tracked, i.e. segmented, for the full 3D bone model to eventually be reconstructed slice-by-slice^32^. A final overall triangular mesh is then exported in a file with the standard stereo-lithography (STL) format, with a huge number of uniform points and triangles, to represent the external cortical surface. This image segmentation is a long and critical process which implies manual, automatic or semi-automatic tracking of the bone silhouettes and, therefore, needs anatomical knowledge, computer skills and awareness of the scopes^33,34^. Many dedicated software packages are available for this, from freeware tools with basic functions^35^, to expensive packages with effective algorithms and features. A best possible software would support the user in accurately defining these 3D bone models, and at the same time would not require extensive manual work from an expert operator; for each different clinical or biomechanical application, a good compromise should be found between automation of the process and quality of the final result^35,36^. Among these software packages for 3D bone reconstruction, Mimics Innovation Suite (Materialise, Leuven, Belgium)—MIS—represents the state-of-the-art for fully supported semi-automatic segmentation of any bone structure, and on the other hand Bonelogic Ortho Foot and Ankle (Disior, Helsinki, Finland)—DIS—represents a most modern tool for fully automatic segmentation of foot bones.

The latter tool includes also automatic calculation of a number of axes embedded into the 3D reconstructed bones, from which absolute and relative orientation angles are calculated. Some of these mimic traditional, i.e. two-dimensional, angular measurements from radiographs^8,9^, others are possible novel representations of foot bone orientations in 3D. This DIS software was developed for medical doctors to analyze medical images of each single patient in 3D, by avoiding the time-consuming bone segmentations and angle calculations^37,38^. It has been recently claimed that these auto-generated 3D measurements of hind- and mid-foot alignments are reliable in both healthy individuals and patients with posttraumatic end-stage ankle osteoarthritis^37^. A similar objective calculation of absolute and relative orientation angles of the foot bones in 3D from current CBCT scans has been proposed recently by the present authors^39^, though that was limited to a single normal foot and only two representative bones. 3D bone models were obtained, and relevant anatomical axes were calculated based on the Principal Component Analysis (PCA), or on landmarks or on mid-diaphyseal shapes. Initial observations on orientation angles of the calcaneus and first metatarsal bone in a simulated sagittal plane projection revealed that the likely malposition of the foot in traditional radiographs can result in angular errors larger than 5 degrees. In 3D measures, this source of error is removed and the inclinations of the bones with respect to the ground plane can be objective, i.e. not dependent on foot positioning and on the operator^40,41^.

3D analyses in weight-bearing have been considered very valuable particularly for the characterisation of flatfoot. An initial study^12^ compared CBCT foot scans from 20 patients with flexible adult acquired flatfoot deformity while standing and seated. These were analyzed in 3D, and in the three anatomical plane projections by three independent observers. In addition to substantial intra-observer and inter-observer reliability, these measurements were observed to differ between weight-bearing and non weight-bearing conditions, with more pronounced foot deformities revealed by the former. In a following paper, apparently on the same clinical population, these measurements in weight-bearing were shown to add significantly also to standard clinical evaluations of the hindfoot alignment^42^. On the same population, the same authors showed later^43^ good correlation between measurements from weight-bearing radiographs and weight-bearing CT; however, significant difference was observed between a number of these measurements, with traditional radiographic assessments underestimating the severity of deformity when compared to 3D CT-based assessments, as it had been observed in previous studies though in pathological feet^40,44^.

The primary scope of the present work is to report original skeletal measurements of the flatfoot finally in 3D and in weight-bearing conditions. The comparison of these measurements from two state-of-the-art tools for the necessary 3D bone model reconstruction, i.e. MIS and DIS, was the secondary scope, still using the same calculations based on the bone anatomical axes. Thus, the present results are expected to contribute both in a better original characterisation of 3D deformities of the flatfoot in weight-bearing, and in the evaluation of semi-automatic versus automatic 3D skeletal reconstruction tools for the quantitative description of foot bone position and orientation.

## Material and methods

### CBCT data collection (Fig. 1A,B)

**Figure 1:**
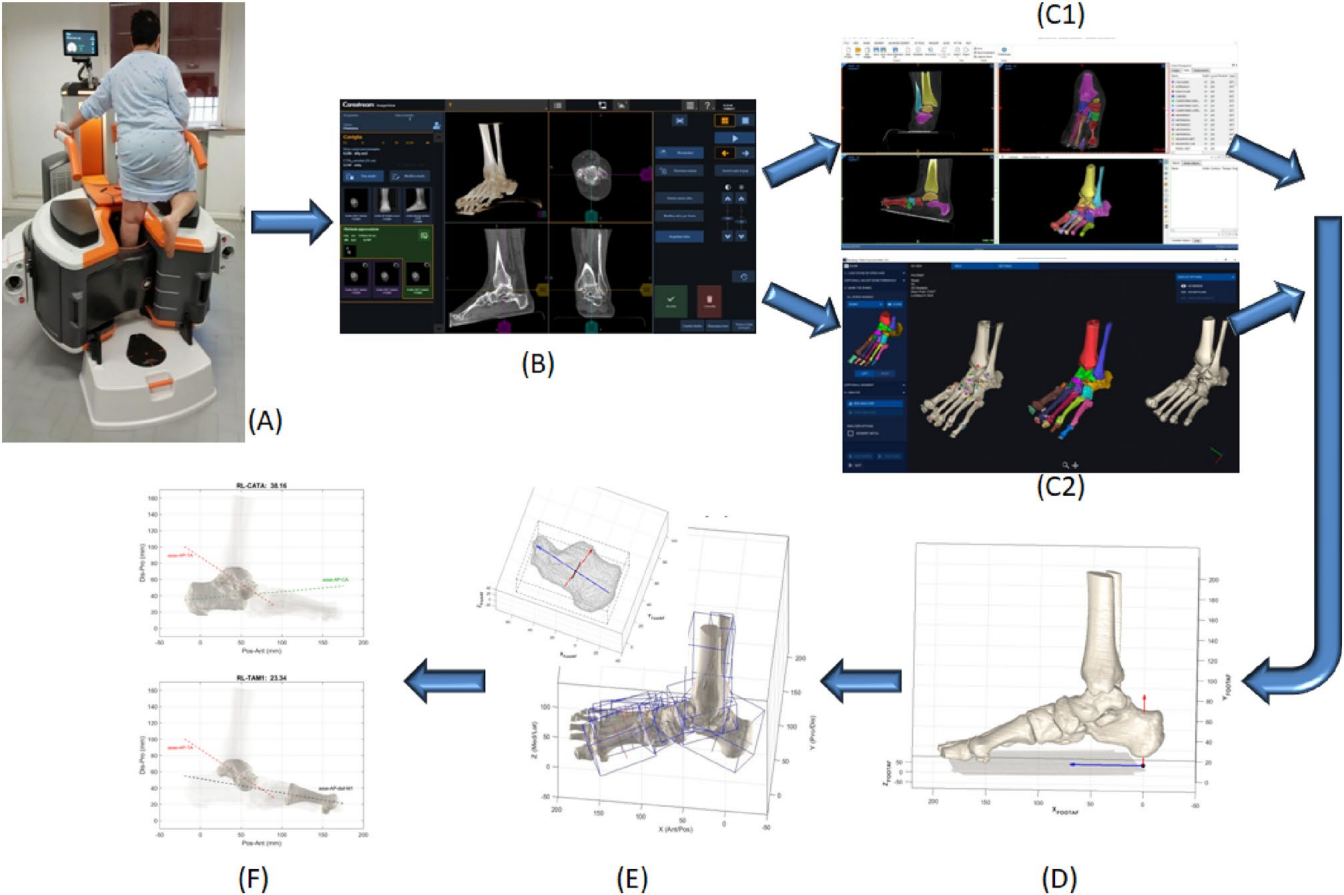
Diagram representing the full process from CBCT scans to bone angular measurements. A typical patient in single-leg weight-bearing during the CBCT scan (**A**). The 3D data-set including volume rendering of the foot is available at the interactive screen (**B**). This Dicom file is used both with the MIS (**C1**) and DIS (**C2**) software, respectively for the semi-automatic and automatic bone segmentation process: all foot and ankle bone segments eventually were modelled separately, and the ground segment is also identified. The same bone models, but in the foot anatomical reference frame (FootAF), here in a lateral view (**D**). Construction of the three anatomical axes by means of the PCA technique (**E**): an exemplary application to the calcaneus model with the three axes depicted (above), and the same for all foot bone models (below). Exemplary diagrams for the calculated angular measurements (**F**): in the lateral projection, the angle between the talus and calcaneus (RL_TACA, above) and between the talus and the 1st metatarsus (RL_TAM1, below), i.e. the Meary’s angle.

21 patients (9 male / 12 female, 50.9 ± 13.6 years old, 79.5 ± 16.2 kg weight, 170.3 ± 11.9 cm height, 27.3 ± 4.0 BMI) with indication for the Mini Bone Block Distraction Subtalar Arthrodesis^45,46^ were analyzed. The affected foot (8 right / 13 left) was scanned in single-leg upright posture, i.e. in weight-bearing, via CBCT (‘OnSight 3D Extremity System’, Carestream, Rochester, NY).

This device has a high performance flat-panel detector and a three-source x-ray tube, to minimize artifacts associated to the in-bore patient motion and the presence of metal. The nominal radiation dose is lower than standard CT devices, accounted for to a single 215 degrees rotation of the tube required for the entire scanned volume. Proprietary algorithms provide 3D volumetric data sets of 884 × 884 × 960 isotropic voxels of 0.26 mm size. The scan time is 25 s, and a 3D rendering of the collected data-set is made available for a visual check in a few minutes (Fig. 1B). For the present scans, x-ray Tube Voltage was set at 90 kVp, the Current at 5.0 mA.

This study received internal review board approval. The investigation was performed in accordance with the relevant guidelines and regulations. Informed consent to participation in this study and to publish relevant anonymized information and images was obtained by all patients. All patients were above the age of 18 years.

### 3D bone model reconstruction by MIS and DIS (Fig. 1C1-C2)

For each foot, from the collected 3D data-set, 960 CT images at 0.26 mm distance were produced in Dicom format. These were processed in MIS (version 22.0), where semi-automatic segmentation of each foot bone was performed by a single operator. A preliminary 3D rendering of the foot model was obtained by setting initial Hounsfield Unit values in the range of 300–500 and 1600–2000, for the lower and upper thresholds respectively. However, in most of the scans, also because of the deformities, the bones were too close to be distinguished, and the density of the bone was low. Thus the bone silhouettes had to be detected and tracked manually by the operator by using the Brush and Lasso tools, and this resulted in a very time-consuming process, both for the refinement of each mask and also for the separation of each bone. The ground was segmented as well, and taken in 3D as the reference transverse anatomical plane for the calculation of the absolute orientation of the bones.

The same Dicom files were used for the same 3D reconstruction according to the DIS (version 1.0.0) technique. This software automatically rendered a 3D isosurface of the bone tissue, where the same operators was required only to place at least one marker point on each visible bone, i.e. labelling. Thereafter, the software automatically registered a mathematical model of each bone on the original rendered image. The software also computed the location of anatomical landmarks and longitudinal axes of the bones of interest^37^, but this feature was not used in the present work.

**Figure 2:**
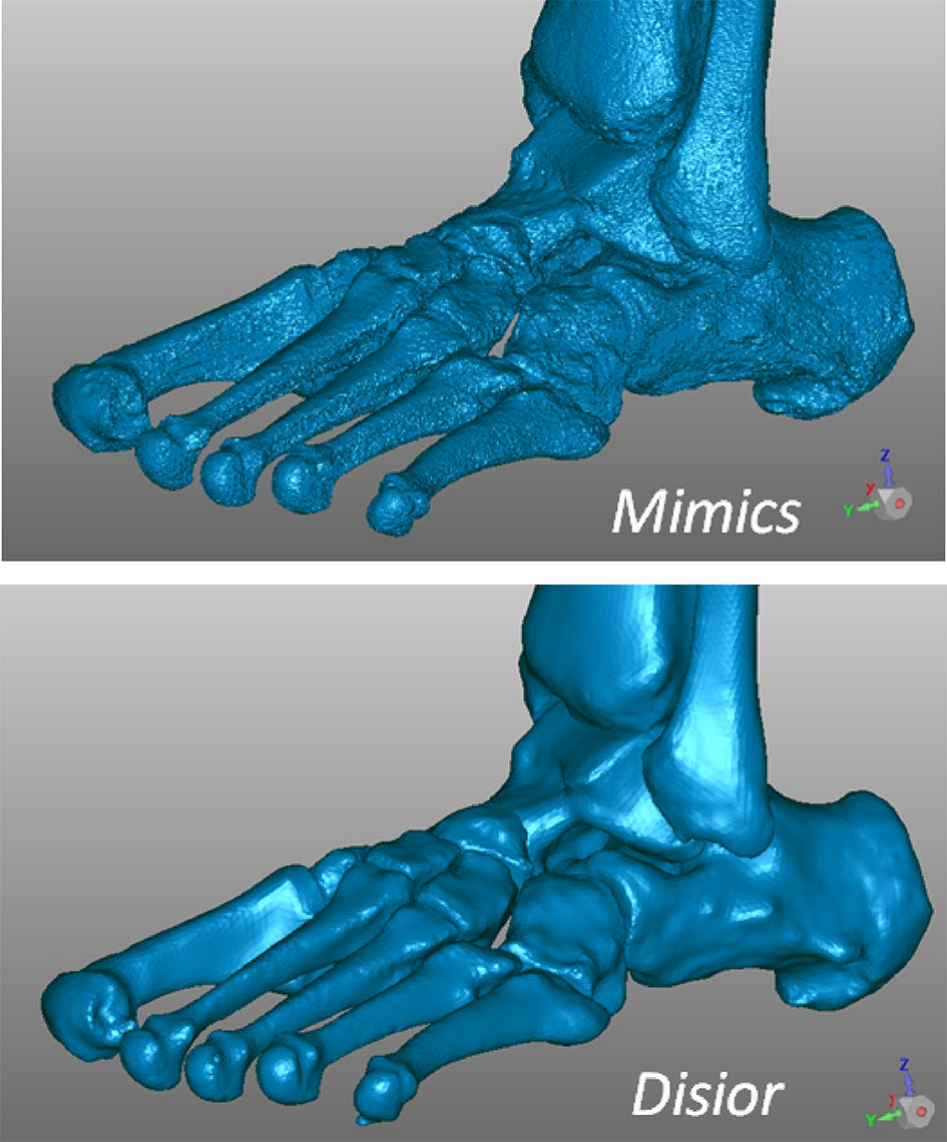
Triangular meshes resulting from the 3D reconstruction of bone models of the same flatfoot, software screenshots from a typical patient of the present study: by using MIS (top) and DIS (bottom). These include distal tibia and fibula and all foot bones but phalanges and sesamoids. The number of points and triangles were respectively 1.137.013 and 2.273.950 in MIS, 140.728 and 281.400 in DIS; the size of the corresponding files was 108 MB in MIS, 13.5 MB in DIS.

Eventually, from both MIS and DIS technique, a 3D model of the cortical shape of each bone is thus obtained in the STL format for each analyzed foot (Fig. 2).

Spatial registration between the two was obtained in Geomagic Control X (3D Systems, Rock Hill, US), just to express them in the same absolute reference frame^29^; in this software the sesamoids and the ground plane were made available also to the DIS-based models.

### Calculation of anatomical planes and bone axes (Fig. 1D-E)

These STL files with the registered bone models were imported in Matlab (Mathworks Inc., Natick, MA, USA) to be processed according to an established procedure^39^. For each entire foot, an anatomical reference frame (FootAF) was first defined with the vertical axis orthogonal to the ground, and the antero/posterior axis as the line segment on the ground plane joining the projections of the most plantar points of the calcaneus and second metatarsal head. All bone models were then realigned in this FootAF, for the following calculations and comparisons to be performed on an anatomical base.

For each bone model, a reference frame with three anatomical axes was defined according to the PCA^39^. Starting from the 3D coordinates of the bone surface points, this statistical analysis searches for the three orthogonal axes with the highest variance. For most of the foot bones, this results automatically, i.e. in a one-shot calculation, in the identification of the longitudinal, medio-lateral and dorsi-plantar anatomical axes. Clearly, this is independent on manual subjective actions or definitions, and on the position and orientation of the bones. These three axes thus represent an anatomical reference frame embedded into each bone, which is always different from the FootAF. These are ultimately 3D axes in the FootAF; in addition, these were also projected into the lateral, frontal or transverse anatomical planes of the FootAF for the following calculations.

### Calculation of absolute and relative bone orientations and heights (Fig. 1F)

**Figure 3:**
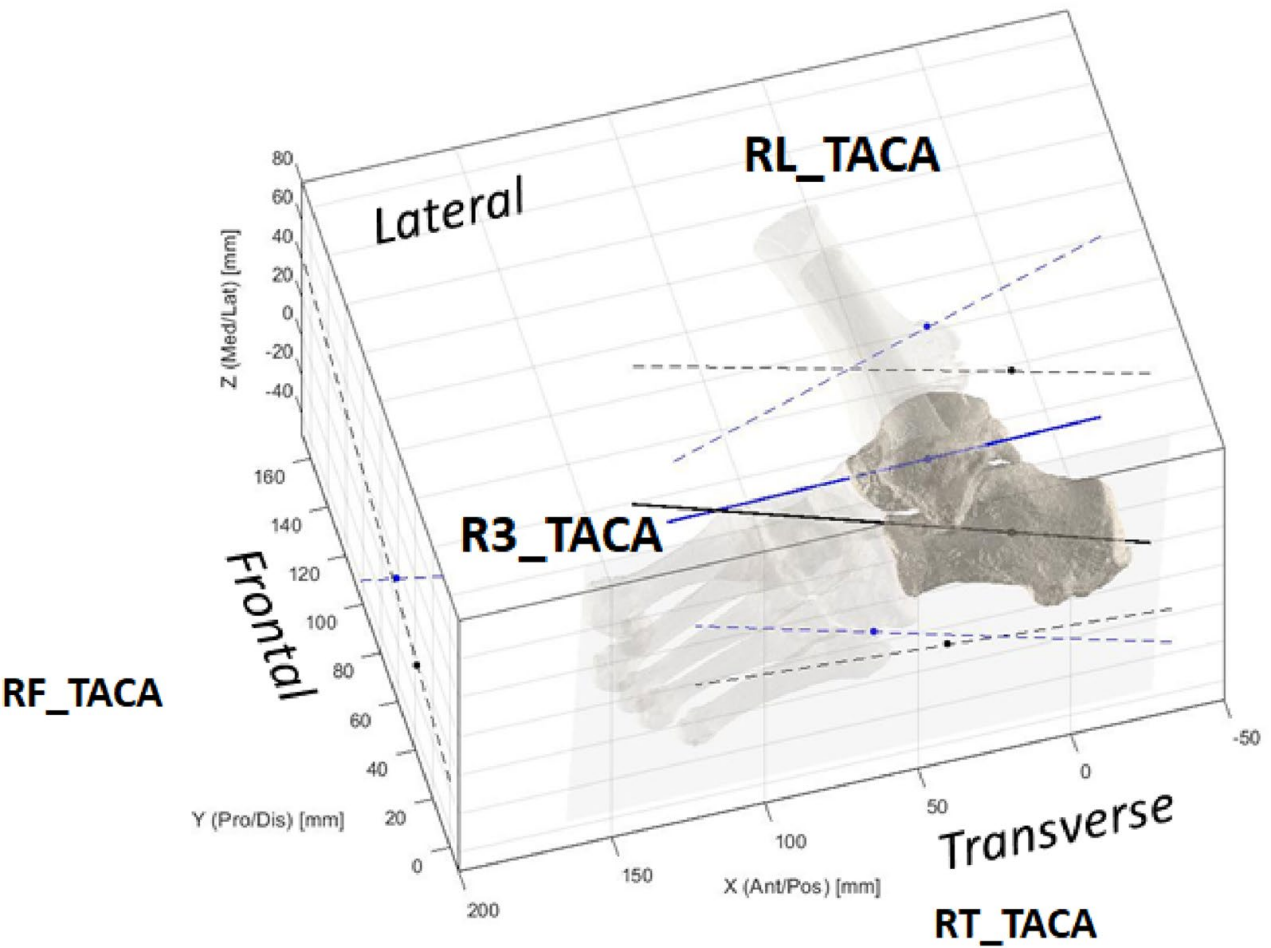
A diagram representing a typical fully 3D calculation of the angles between the longitudinal axes of the talus and calcaneus (‘TACA’): the three projections on the anatomical planes (RL_ RF_ RT_), and the single overall 3D calculation (R3_).

The last processing step, i.e. angular and height measures (see also Table 1) were divided into absolute inclination (I) of a single bone, or relative orientation (R) between two adjacent bones^39,47^. Both were further divided in 3D measures (3), or planar measures in the lateral (L), frontal (F) or transverse (T) anatomical planes, similarly to what defined traditionally in foot radiographs^8,9^. Height of the bone with respect to the ground (Hg) was also calculated as minimum distance, i.e. the height of the most plantar point of the bone model. These measures were applied to the following bones: tibia (TI), calcaneus (CA), talus (TA), navicular (NA), cuboid (CU), medial cuneiform (CM), and 1st and 2nd metatarsal (M1, M2). With this nomenclature and notations, all angular measures have a unique simple acronym. For example I3_CA is the 3D inclination of the calcaneus, i.e. calcaneal pitch; RT_TACA is the angle between the talus and calcaneus in the transverse plane projection (Fig. 3); RL_CAM1 is the angle between the calcaneus and the 1st metatarsus in the lateral plane projection, i.e. the Hibb angle^39^; RL_TAM1 is the angle between the talus and the 1^st^ metatarsus in the lateral plane projection, i.e. the Meary’s angle (Fig. 1F). The Foot and Ankle Off-set (FAO) was also calculated. Eventually, according to this nomenclature, the following 32 measurements were taken from the foot bone models, from both MIS and DIS techniques:

**Table 1:** List of the 32 original measures, with Units, Descriptions and relevant Acronyms.

Code	Unit	Description	Measurements	N
I	Deg	Single bone inclinations	IL_TI , IL_TA, IL_CA, IT_TA, IT_CA	5
R	Deg	Relative bone angles	RF_TICA, RL_TICA, RT_TICA, RF_TACA, RL_TACA, RT_TACA, RF_TANA, RL_TANA, RT_TANA, RL_CAM1, RL_TACAM1, RL_TAM1, RT_TAM1, RT_M1M2	14
3	Deg	3D angular measurements	I3_CA, I3_TA, R3_TICA, R3_TACA, R3_CAM1, R3_TAM1, R3_TANA, R3_TACAM1	8
H	mm	Heights from the ground	Hg_NA, Hg_CU, Hg_CM	3
FAO		Foot–ankle offset related measurements	FAO_%, FAO_mm	2
Total				32

### Statistical analyses

According to current conventions in biomedical sciences, a power of 80% and an α level of 0.05 was defined, which resulted in a sample size of at least 16 feet to report significant differences between MIS and DIS based calculations. This computation assumes that the general mean difference was 1.0° and 1.0 mm in angular and linear evaluations, and the relevant common within-group standard deviation was 1.0° and 1.0 mm, respectively. This was eventually found in the present results. The Kolmogorov–Smirnov test was first applied to check for normal data distribution; accordingly, Student’s paired-t test or Mann–Whitney-Wilcoxon test was used for comparison between MIS and DIS based measurements. Furthermore, the Pearson product–moment correlation coefficient (r) and its squared form (r^2^), i.e. the coefficient of determination, were also used to derive correlations, for both MIS and DIS analyses. Corresponding p-values were reported for assessing significance in terms of differences and correlations, this being accepted at *p* < 0.05. These statistical analyses were performed in Matlab (The MathWorks, Inc., Natick, MA, USA).

### Ethical approval

The study was approved by the ethical committee of the IRCCS Istituto Ortopedico Rizzoli, Bologna—Italy (Prot. Gen 0,012,502, 5th November 2018). The authors certify that the institution approved the investigation protocol, that all investigations were conducted in conformity with ethical standard of research. In detail, the investigation methodology was performed in accordance with the relevant guidelines and regulations. Informed consent for participation in this study and to publish related anonymized information/images was obtained by all patients. All patients were above the age of 18 years.

### Consent to participate

Signed Informed consent for participation in this study and to publish related anonymized information/images was obtained by all patients. All patients were above the age of 18 years.

## Results

The severity of the foot deformity was revealed by many measurements (Table 2), involving different bones, both in two-dimensional projections and in 3D. This can be observed in the inclination in the lateral plane of the talus and calcaneus, i.e. IL_TA and IL_CA, and the accompanied collapse of the fore-foot with respect to the talus, or the Meary’s angle, i.e. RL_TAM1. Clearly, the medial longitudinal arch is affected, here represented as the Calcaneal-to-1^st^metatarsal angles, or the Hibb angle, i.e. RL_CAM1, and the Moreau-Costa-Bertani, i.e. RL_TACAM1. These two measures were found 19 degree different in the lateral projection, 24 degree different in 3D, in both MIS- and DIS-based models. For both angle definitions, and for both MIS- and DIS-based models, 3D values showed smaller standard deviations than the corresponding lateral plane projections. Distance from the ground of the navicular, cuboid and medial cuneiform were found to be consistent over the feet, with a standard deviation of about 20% of the mean values; these distances were not found more consistent after normalisation using the foot length.

**Table 2:** Mean and standard deviation (SD) of the 32 measurements across the patients, for both MIS- and DIS-based calculations.

	MIS		DIS	
	Mean	SD	Mean	SD
IL_TI*	8.94	5.93	8.36	5.76
IL_TA*	24.33	7.49	20.88	7.28
IL_CA*	− 8.83	5.11	− 7.77	5.02
IT_TA	27.38	10.55	27.00	10.70
IT_CA*	5.91	4.05	5.18	3.78
RF_TICA	49.43	24.73	51.03	29.09
RL_TICA*	17.59	7.13	16.00	6.72
RT_TICA*	59.55	22.44	57.18	24.33
RF_TACA*	86.34	27.45	93.09	33.79
RL_TACA*	33.16	6.53	28.64	6.22
RT_TACA	− 21.47	10.71	− 21.82	10.20
RF_TANA	74.29	12.34	75.92	13.61
RL_TANA*	13.10	12.04	16.29	11.76
RT_TANA*	− 48.16	13.12	− 45.79	12.88
RL_CAM1*	158.87	8.63	159.74	8.36
RL_TACAM1*	139.71	8.37	140.28	7.99
RL_TAM1*	− 12.12	8.79	− 8.50	8.97
RT_TAM1	− 20.38	11.23	− 20.80	12.12
RT_M1M2*	− 13.09	3.66	− 12.38	3.29
FAO[%]	13.32	4.32	13.36	4.43
FAO[mm]	19.45	6.68	19.52	6.77
I3_CA*	− 8.77	5.09	− 7.73	5.01
I3_TA*	21.29	5.65	18.18	5.43
R3_TICA*	73.95	6.97	75.15	6.74
R3_TACA*	37.54	8.11	34.45	8.07
R3_CAM1	157.77	8.17	158.33	7.55
R3_TAM1	22.93	9.81	22.58	10.35
R3_TANA	45.60	8.93	45.24	8.89
R3_TACAM1	133.65	6.61	133.88	6.28
Hg-NA*	24.22	5.24	23.97	5.30
Hg-CU*	18.71	3.57	18.51	3.57
Hg-CM*	18.55	3.39	18.46	3.40

Statistically significant (*p* < 0.05) differences between these calculations are marked with *.

‘I’ and ‘R’ measurements in degrees, ‘H’ in millimetres.

**Figure 4:**
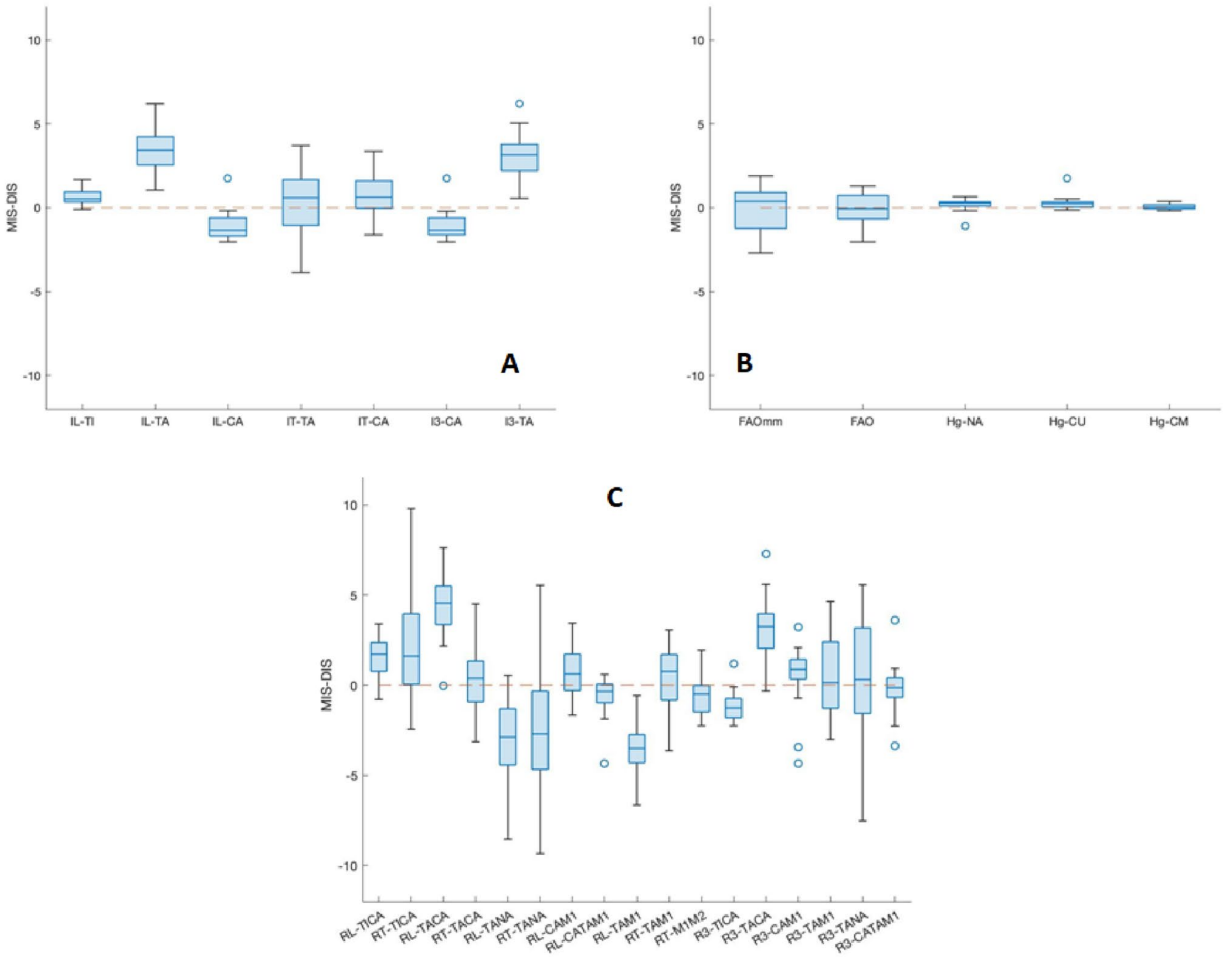
Boxplots with relevant outliers, of the difference between the measurements based on MIS and DIS bone models: the closer to 0 are the plots, the smaller are the differences. These are shown for the single bone inclinations (**A**; in degrees), for FAO and distances from the ground (**B**; mm apart the FAO in %,), and relative bone angles (**C**; in degrees). In each plot, the boxes have lines at the lower, median, and upper quartile values over the analysed patient population. The whisker lines extending from each box show the total data extent; outlier values are reported beyond the whisker ends.

As for the secondary scope of the study, very small differences were found between MIS- and DIS-based measurements (Table 2, Fig. 4). Significant linear correlation between the two tools was found for all these measurements. Statistically significant difference was not found for the following 11 measurements: IT_TA, RF_TICA, RT_TACA, RF_TANA, RT_TAM1, R3_TAM1, R3_TANA, R3_CAM1, R3_TACAM1, and FAO_% and FAO_mm. Rather a large MIS to DIS difference was experienced in procedural time, with about a 10-to-1 ratio.

In general, statistically significant correlations were found between the measurements (Table 3). For example, for RL_TACA with IL_CA IT_TA RT_TACA RL_TANA RT_TANA RT_TAM1 I3_TA R3_TACA R3_TAM1 (range of R^2^ 0,21 ÷ 0,81). Interesting correlations were found also for RT_M1M2 with RF_TANA RT_TANA R3_TANA (R^2^ 0,19 ÷ 0,24), and for FAO % with IT_CA RL_CAM1 RL_TACAM1 RL_TAM1 (i.e. Meary) R3_TICA R3_CAM1 Hg_NA Hg_CM (R^2^ 0,20 ÷ 0,47).

**Table 3:** r-values of the correlations between all measurements.

	IL_TI	IL_TA	IL_CA	IT_TA	IT_CA	RF-TICA	RL_TICA	RT_TICA	RF_TACA	RL_TACA	RT_TACA	RF_TANA	RL_TANA	RT_TANA	RL_CAM1	RL_TACAM1
IL_TI		0.32	0.22	0.31	0.38	0.34	0.71*	− 0.90*	0.40	0.20	− 0.16	0.28	− 0.27	− 0.37	0.16	0.18
IL_TA			0.52*	0.72*	0.26	0.49*	− 0.10	− 0.30	0.44*	0.74*	− 0.61*	0.23	− 0.83*	− 0.64*	0.27	0.52*
IL_CA				0.29	0.23	0.82*	− 0.53*	− 0.24	0.71*	− 0.19	− 0.20	0.24	− 0.60*	− 0.35	0.86*	0.92*
IT_TA					0.15	0.30	0.09	− 0.41	0.54*	0.60*	− 0.93*	0.55*	− 0.59*	− 0.76*	− 0.02	0.15
IT_CA						0.64*	0.15	− 0.22	0.62*	0.12	0.23	0.36	− 0.41	− 0.29	0.27	0.31
RF-TICA							− 0.30	− 0.29	0.91*	− 0.08	− 0.05	0.28	− 0.66*	− 0.31	0.73*	0.79*
RL_TICA								− 0.63*	− 0.16	0.30	− 0.03	0.10	0.20	− 0.09	− 0.47*	− 0.51*
RT_TICA									− 0.42	− 0.16	0.32	− 0.39	0.28	0.44*	− 0.12	− 0.11
RF_TACA										− 0.06	− 0.30	0.50*	− 0.61*	− 0.47*	0.56*	0.61*
RL_TACA											− 0.54*	0.08	− 0.48*	− 0.46*	− 0.36	− 0.13
RT_TACA												− 0.41	0.43	0.64*	0.12	− 0.03
RF_TANA													− 0.33	− 0.82*	0.23	0.18
RL_TANA														0.45*	− 0.40	− 0.61*
RT_TANA															− 0.24	− 0.31
RL_CAM1																0.88*
RL_TACAM1																
RL_TAM1																
RT_TAM1																
RT_M1M2																
FAO_%																
FAO_mm																
I3_CA																
I3_TA																
R3_TICA																
R3_TACA																
R3_CAM1																
R3_TAM1																
R3_TANA																
R3_TACAM1																
Hg_NA																
Hg_CU																
Hg_CM																

	RL_TAM1	RT_TAM1	RT_M1M2	FAO_%	FAO_mm	I3_CA	I3_TA	R3_TICA	R3_TACA	R3_CAM1	R3_TAM1	R3_TANA	R3_TACAM1	Hg_NA	Hg_CU	Hg_CM
IL_TI	− 0.30	− 0.18	− 0.23	− 0.14	− 0.12	0.23	0.27	− 0.68*	0.14	0.24	0.19	0.22	0.30	− 0.10	− 0.10	0.16
IL_TA	− 0.80*	− 0.70*	− 0.32	0.15	0.13	0.52*	0.96*	0.13	0.75*	0.30	0.75*	0.33	0.38	− 0.53*	0.04	− 0.08
IL_CA	− 0.71*	− 0.30	− 0.23	0.39	0.43	1.00*	0.48*	0.54*	− 0.05	0.86*	0.48*	0.18	0.76*	− 0.59*	− 0.39	− 0.42
IT_TA	− 0.41	− 0.88*	− 0.32	− 0.22	− 0.26	0.29	0.53*	− 0.08	0.82*	0.09	0.79*	0.57*	0.24	− 0.19	− 0.28	0.20
IT_CA	− 0.36	− 0.21	− 0.25	0.45*	0.49*	0.24	0.28	− 0.02	− 0.09	0.31	0.33	0.13	− 0.06	− 0.21	0.15	− 0.05
RF- TICA	− 0.67*	− 0.34	− 0.26	0.40	0.46*	0.82*	0.45*	0.37	− 0.07	0.73*	0.53*	0.11	0.54*	− 0.46*	− 0.20	− 0.24
RL_TICA	0.26	0.05	− 0.04	− 0.42	− 0.43*	− 0.52*	− 0.12	− 0.99*	0.17	− 0.40	− 0.17	0.07	− 0.28	0.34	0.15	0.45*
RT_TICA	0.23	0.21	0.42	0.17	0.20	− 0.24	− 0.22	0.64*	− 0.22	− 0.23	− 0.19	− 0.29	− 0.21	0.11	0.22	− 0.19
RF_TACA	− 0.51*	− 0.51*	− 0.35	0.20	0.23	0.72*	0.32	0.22	0.07	0.61*	0.61*	0.31	0.47*	− 0.32	− 0.32	− 0.09
RL_TACA	− 0.36	− 0.57*	− 0.19	− 0.13	− 0.18	− 0.18	0.73*	− 0.28	0.90*	− 0.33	0.49*	0.23	− 0.16	− 0.15	0.35	0.24
RT_TACA	0.27	0.78*	0.22	0.38	0.44*	− 0.19	− 0.42	0.07	− 0.84*	0.02	− 0.66*	− 0.51*	− 0.25	0.11	0.33	− 0.22
RF_TANA	− 0.29	− 0.40	− 0.49*	0.27	0.24	0.24	0.10	− 0.08	0.23	0.36	0.44*	0.78*	0.00	− 0.44*	− 0.44*	− 0.15
RL_TANA	0.74*	0.68*	0.35	− 0.37	− 0.36	− 0.60*	− 0.82*	− 0.24	− 0.52*	− 0.41	− 0.77*	− 0.07	− 0.28	0.55*	− 0.07	0.05
RT_TANA	0.57*	0.57*	0.49*	− 0.17	− 0.13	− 0.35	− 0.50*	0.07	− 0.56*	− 0.37	− 0.61*	− 0.90*	− 0.22	0.55*	0.39	0.21
RL_CAM1	− 0.74*	− 0.04	− 0.10	0.56*	0.64*	0.86*	0.28	0.50*	− 0.33	0.98*	0.34	0.13	0.69*	− 0.67*	− 0.46*	− 0.63*
RL_TACAM1	− 0.78*	− 0.25	− 0.15	0.57*	0.63*	0.92*	0.53*	0.55*	− 0.10	0.85*	0.49*	0.14	0.73*	− 0.75*	− 0.27	− 0.62*
RL_TAM1		0.45*	0.23	− 0.46*	− 0.50*	− 0.72*	− 0.79*	− 0.30	− 0.32	− 0.74*	− 0.69*	− 0.30	− 0.57*	0.77*	0.21	0.46*
RT_TAM1			0.00	0.02	0.04	− 0.30	− 0.56*	− 0.08	− 0.75*	− 0.12	− 0.94*	− 0.33	− 0.18	0.20	0.25	− 0.16
RT_M1M2				− 0.20	− 0.10	− 0.23	− 0.28	0.07	− 0.22	− 0.15	− 0.04	− 0.44*	0.07	0.41	− 0.15	0.19
FAO_%					0.98*	0.39	0.25	0.47*	− 0.26	0.53*	0.21	0.09	− 0.07	− 0.69*	0.02	− 0.65*
FAO_mm						0.44*	0.24	0.50*	− 0.33	0.60*	0.22	0.04	0.02	− 0.68*	− 0.01	− 0.64*
I3_CA							0.48*	0.54*	− 0.05	0.86*	0.48*	0.18	0.76*	− 0.59*	− 0.38	− 0.42
I3_TA								0.16	0.66*	0.27	0.64*	0.16	0.31	− 0.54*	0.21	− 0.09
R3_TICA									− 0.19	0.43	0.22	− 0.08	0.28	− 0.35	− 0.13	− 0.45*
R3_TACA										− 0.27	0.62*	0.36	− 0.05	− 0.12	0.08	0.30
R3_CAM1											0.39	0.26	0.68*	− 0.66*	− 0.54*	− 0.61*
R3_TAM1												0.35	0.32	− 0.44*	− 0.32	− 0.09
R3_TANA													0.13	− 0.41	− 0.47*	− 0.30
R3_TACAM1														− 0.40	− 0.48*	− 0.37
Hg_NA															0.15	0.78*
Hg_CU																0.21
Hg_CM																

Statistically significant (*p* < 0.05) values are marked with*.

## Discussion

The present study shows that CBCT and 3D bone reconstruction techniques can provide valuable assessment of the complex foot bone deformities finally in weight-bearing, eliminating the previous limitations of unloaded feet and bidimensional angular measurements. Bone shapes were reconstructed by using two very different segmentation tools available in the market, and the corresponding measurements here compared well. The following paragraphs discuss and justify this work and these material and methods, as well as the relevant literature.

The adult acquired flatfoot is a very common disease and appears as a complex osseous derangement. It is known as a biomechanical 3D effect of deforming forces, tendon dysfunction, ligament disruption, and joint subluxation, which results in the overall loss of dynamic and static function of the foot^48^. A careful clinical examination should imply a thorough radiographical analysis^49^, possibly in 3D and in weight-bearing^50^, particularly to support the surgeon to identify the joints that are most unstable^51^, to monitor the progression of the deformities on follow-up^10^, to plan the complex surgical procedures necessary to correct the malalignments^52^, and to assess quantitatively the 3D effects of the surgical interventions^53^. New instruments and techniques are now available finally for these analyses to be performed with modern tools and limited efforts^13,20,21,39^. These tools are expected to show finally the true orientation of bones and joints of the foot during physiological loading^7^. The reliability and the limitations of these tools should be tested more carefully, particularly by exploiting these within the complex context of adult acquired flatfoot. In the present work, for the first time a number of angular and linear measurements were taken on these patients fully in 3D and in weight-bearing. In many previous similar papers from CBCT scans in fact the angles were taken still in planar views, i.e. in the most appropriate single CT image^10,17,19,37,40,42–44,54,55^ and not exactly from 3D models of the foot bones as in the present work. The entire process here present (Fig. 1) is also fully automatic, apart from the likely manual detection of the bone silhouettes in the CT slices by using MIS (Fig. 1C1), as discussed above.

The present measurements in weight-bearing feet include original planar and 3D angles, in addition to distances. For planar angles, the longitudinal axis of a bone in 3D, or of a couple of bones, is projected in one of the three anatomical planes, and the relevant inclination angle with respect to the ground, or with respect to the other bone, is calculated, similarly to traditional X-ray based angular measurements^8,9^. 3D angles are also calculated originally in the present study, still based on the same 3D bone models, according to what proposed recently by these authors^39^. For single bones, the angle between the longitudinal axis and the ground is taken in the 3D plane containing this axis and its projection into the ground. For couples of bones, mathematics allow the calculation of a 3D spatial angle between their longitudinal axes. With the present full 3D technique, objective and comprehensive angular and linear measurements finally can be taken. These are based on unique and repeatable anatomical axes and planes, thus removing by definition any source of undesired variability associated to the radiological apparatus, foot positioning with respect to the projection planes, and subjective identification of landmarks. The present anatomical axes in the 3D space in fact, being based on the automatic PCA statistical procedure and the entire mesh of the bone, are a robust representation of the full bone shape. Real 3D angles are here represented, independent also by the deformity of the foot, which implies different projections on the same image plane^39^. The present planar angles can be compared to all previous traditional foot angular measurements, but the present 3D angles at the moment do not find equivalents for a reasonable comparison with the literature.

The present thorough 3D measurements demonstrate further the large deformities implied in severe flat feet, involving many bones and the three anatomical planes, in agreement with similar observations from the literature, though most of these were taken with traditional x-ray projections^8,51,56^. The present population of 21 patients was found overweight, with BMI over 30 in 6 patients, and smaller than 25 in only 6 patients. There were concerns on the consistency of the leg position during the scans in weight-bearing, but the tibia in the lateral projection was found inclined (IL_TI) in dorsiflexion from 0 to 23 degrees, consistent with the overall single leg up-right position and the subjective comfortable postures. In general, the 3D measurements were more consistent than those obtained with planar projections, demonstrating the issues associated to the latter, as discussed. For example, talus and calcaneus in 3D (I3_TA, I3_CA) showed much smaller standard deviations than in the corresponding projections into the lateral (IL_TA, IL_CA) and transverse (IT_TA, IT_CA) planes. Also the complex talonavicular joint is represented more consistently in 3D (R3_TANA) than in any of the three anatomical plane projections (RF_ TANA, RL_ TANA, RT_TANA). The same applies also to the 1^st^ metatarsus with respect to the talus, with R3_TAM1 much more consistent than RL_TAM1 and RT_TAM1. The medial longitudinal arch, very relevant in the flatfoot and represented in a number of different ways^9^, is here reported both as the Calcaneal-to-1^st^metatarsal angle^57^ and as the Moreau-Costa-Bertani angle^58^, both in the lateral plane projection and in 3D. The mean values were found very different, demonstrating a considerable bias between these two definitions, though small was the difference between planar projection and 3D measurements.

Two software were here exploited for the 3D bone model reconstruction, and these demonstrated their claimed features. MIS has a large series of thorough tools for manual and semi-automatic segmentation, which usually results in very detailed and accurate bone models. DIS has the fastest and fully automatic algorithm for the same scope, and thus the results are fully independent on the operator, and also smoother than those from MIS; however, its reliability has been assessed only on the cranium^38^. The present investigation did not want to investigate in detail the differences between MIS-based and DIS-based bone models in themselves, but rather to show the extent to which the angular measurements calculated from these models do compare well or not. The results also show that the standard deviation of the 32 measurements over the present population is smaller with DIS than with MIS. As expected, the 3D models of the foot by MIS are the most realistic, also with small geometrical details, more suitable for careful analyses of bone impingements, joint space, distance map, and morphological bone deformations over time, as well as in case of biomechanical modelling, pre-surgical planning, and patient-specific instrumentation design. On the other hand, DIS tool is much more efficient in terms of time-consuming: in the present analysis, on average the 3D reconstruction of all foot bones took about 90 min by using DIS, and about 15 h in a beginner and 10 h in an expert by using MIS. This time of course depends largely on a number of factors, mainly the target accuracy, the necessary details, the quality of the scans, and particularly the bone density^59^. Segmentation time can be in any case reduced considerably in both software if only a few measurements in a limited number of bones are meant to be analyzed.

The present study has limitations. With the present CBCT device not the entire foot is scanned, because of the size of the field-of-measurement; however only part of the phalanges were lost in the present study. The bone reference frames were calculated automatically via the PCA technique, which represents anatomical axes not in the traditional way for some foot bones; in particular the varus/valgus of the calcaneus (RF_TICA) (see also^42,60^) and of the talonavicular (RF_TACA) articulations deserve further definitions and analyses, more careful and more clinically oriented. In a few feet substantial differences were observed between the MIS and DIS-based angular measurements; a cautious analysis on the corresponding 3D bone models from the two segmentation software should be performed in the future. However this was not within the scopes of the present work, and just suggests deeper relevant analyses in the future. All the present measurements have only a few established reference values in the literature, because of the very original calculations here performed fully in 3D and in weight-bearing. In particular the 3D angular measurements between two bones, i.e. R3, has also a difficult geometrical interpretation, but has the advantage to represent in a single value the relative position between two bones in the three anatomical planes. Thorough comparisons with corresponding measurements from a control population, i.e. asymptomatic feet, would be necessary, but this is critical also for the ethical issues associated to radiation doses. The present analysis was performed in patients before a surgical correction procedure, thus in a severe stage of the pathology, where large osseous derangements are expected. Clearly, because of the present time-consuming overall process for 3D skeletal measures, i.e. bone model reconstruction by either MIS or DIS tool, PCA and angle calculations (Fig. 1), these hardly can be introduced in routine clinical assessments, which necessarily need a quantitative response in a few minutes; rather, qualitative views of the 3D rendering of the feet can be accessible in a few seconds^31^. On the other hand, the present 3D measurements can be exploited easily in large clinical populations in case of retrospective and prospective clinical studies, as well as in complex biomechanical analyses^13,47^.

In the future, additional original measurements can be established, taking full advantage of the 3D nature of the CT scans in weight-bearing, i.e. CBCT. More careful reconstructions of foot bone models and definitions of relevant anatomical frames shall be identified, with the scope to remove the anomalies associated to the asymmetry of some of these bones, such as the talus and calcaneus. Novel volumetric analyses of the syndesmosis or of the three arches of the foot may overcome their traditional simplified geometrical representations. Distances from the ground or between bones can be calculated more thoroughly using the centroid of the corresponding models. Traditional and novel measurements in 3D can be used for thorough assessments before and after operation^61^. In addition to the effective Foot–Ankle-Offset^62,63^, other new indexes representing the overall foot deformities in a single value shall be defined, to support better the clinical decision making process. Also the current multi-planar measures of articular subluxations^19^ can be ameliorated with complete 3D bone models. All these advancements may also be supported by modern visualisation modalities, for example from virtual-reality or 3D printing. The present techniques and measures shall be combined with other medical imaging^23^ and biomechanical^13,47,56,64^ analyses, for a thorough 3D radiological and functional assessments of the severity of the pathology and of the effects of treatments. Finally, distance map analyses can detect very carefully how the disruption of the skeletal structure affects the joint surface interactions under load^65^.

The modern weight-bearing CT scans together with the present 3D bone reconstruction and angular measurement techniques allow thorough measurements to quantify accurately flatfoot deformity. Traditional planar and novel 3D angles of the foot bones from relevant models confirm the severe overall osseous derangement. Both the MIS and DIS software transform carefully medical images into 3D bone models, from which angular and linear measurements can be extracted, thus providing foot and ankle physicians with quantitative measures for clinical diagnosis, treatment planning and outcome assessment. Most of the present measurements are fully automatic, and not affected by human errors in foot orientation and projection artefacts, thus overcoming traditional measures from planar radiographs.

## Data Availability

The datasets used and/or analyzed during the current study are available from the corresponding author on reasonable request.
